# Chest imaging findings in COVID-19 patients: a case series from Nigeria

**DOI:** 10.11604/pamj.2020.37.39.25006

**Published:** 2020-09-09

**Authors:** Olubukola Khadija Ajiboye, Oludolapo Sherifat Katibi, Ohiole Ayeni, Chiedozie Osuoji, Olubusayo Abimbola Agbaje

**Affiliations:** 1 Radiology Department, Cedarcrest Hospitals, Abuja, Nigeria,; 2 Department of Infection, Immunity and Cardiovascular Disease, University of Sheffield, Sheffield, United Kingdom,; 3 Emergency Department, Cedarcrest Hospitals, Abuja, Nigeria,; 4 Radiology Department, Wellmed Healthcare Limited, Lagos, Nigeria

**Keywords:** COVID-19, imaging, chest, Nigeria

## Abstract

COVID-19 is a global pandemic ravaging the whole world with large numbers of reported cases globally. It is a highly-contagious novel infectious disease that causes inflammation in the respiratory system. Chest imaging is a useful adjunct for diagnosis, documenting the extent of disease as well as observation of changes and is thus, strongly recommended in suspected COVID-19 cases, for initial evaluation, differential diagnoses and follow-up. Description of typical imaging findings abound worldwide with a dearth of similar publications in sub-Saharan Africa. This series documents the chest imaging findings from a single facility of four cases between the ages of 38 and 60 who all tested positive for COVID-19 with real-time, reverse transcriptase polymerase chain reaction of the nasopharyngeal swabs.

## Introduction

COVID-19 is a novel infectious disease caused by a type of RNA virus, belonging to the family of coronaviruses, leading primarily to a pneumonia [1]. It was first reported in Wuhan China in December 2019 and has since spread to all continents, becoming a global pandemic. The African continent initially lagged behind the rest of world to record an upsurge of cases; however, more cases are now being reported in Africa with Nigeria and South Africa leading the pack. As of June 29^th^, 2020, Nigeria had 29,286 documented cases with 16,804 active cases [2]. Chest imaging is mainly by computed tomography (CT) supplemented by radiography [3]. These can be done quickly and non-invasively and provides insight for diagnosis, evaluation of severity, complications and disease-progression [3]. This is a report of chest imaging of four cases positive for COVID-19 in a private facility in Nigeria, highlighting the role of imaging in the diagnosis and management of suspected cases in health facilities in Nigeria.

## Methods

This was a retrospective review of four cases, with an age range of 38 to 60 years. They were seen in our hospital in Abuja, Nigeria, between 1^st^ April to 18^th^ June 2020 with features suggestive of pneumonia and they had a positive real-time polymerase chain reaction (RT-PCR) test for COVID-19. The COVID-19 confirmatory tests on nasopharyngeal swabs were carried out at the National Reference Laboratory, Abuja through the real-time polymerase chain reaction. We examined the clinical presentations, laboratory and imaging findings in these adults.

## Results

**Figure 1:**
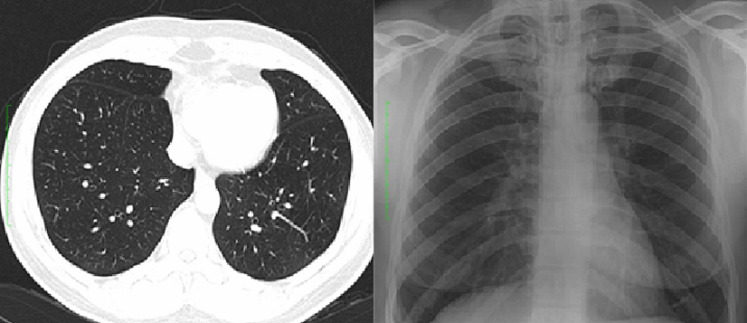
chest radiograph and CT images of case 1 on the 5^th^ and 19^th^ days of onset of symptoms respectively showing no evidence of pneumonia

**Case 1:** presented in our outpatient clinic with complaints of difficulty in breathing, which began 5 days prior, associated with dizziness. There was no history of cough or chest pain or fever, He had a sample taken for COVID-19 just prior to presentation, which turned out to be positive and was admitted to the isolation centre. He had no known comorbidities. No abnormality was found on clinical examination. His chest X-ray (CXR) showed no evidence of pneumonia. He represented two weeks later following discharge from an isolation centre on account of chest pain, difficulty in breathing and fatigue. A computerised tomography (CT) of the chest also revealed no abnormalities. Full blood count, C-reactive protein (CRP) were normal. He was reassured and no further care was given (Figure 1).

**Figure 2:**
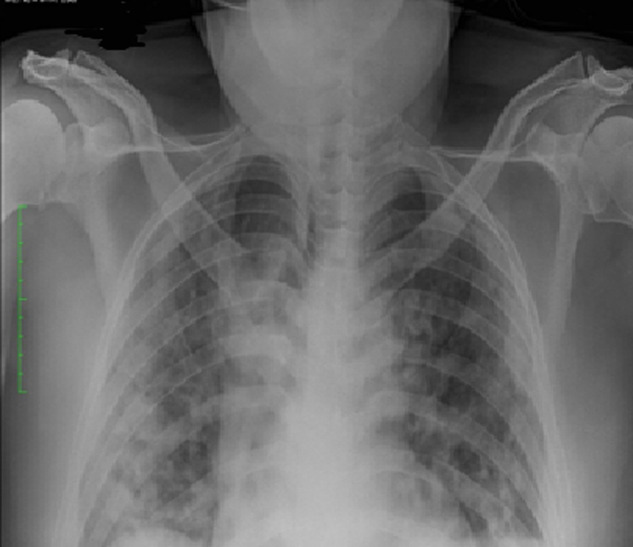
chest radiograph of case 2 on the 5^th^ day of onset of symptoms, showing bilateral nodular opacities with a peripheral and predominantly lower lung zone distribution

**Case 2:** is a known diabetic with poor glycaemic control admitted via the emergency room on account of cough and difficulty in breathing of 5 days duration as well as diarrhoea. He was afebrile and had reduced air entry in both lungs. He had hypoxaemia improving on oxygen therapy and on lying prone. His radiograph showed bilateral, nodular opacities with a peripheral and predominantly lower lung zone distribution (Figure 2). His full blood count was normal, d-dimer and CRP were elevated, liver function tests deranged. His COVID-19 RT-PCR test results were positive and he was evacuated to the isolation centre for further management.

**Figure 3:**
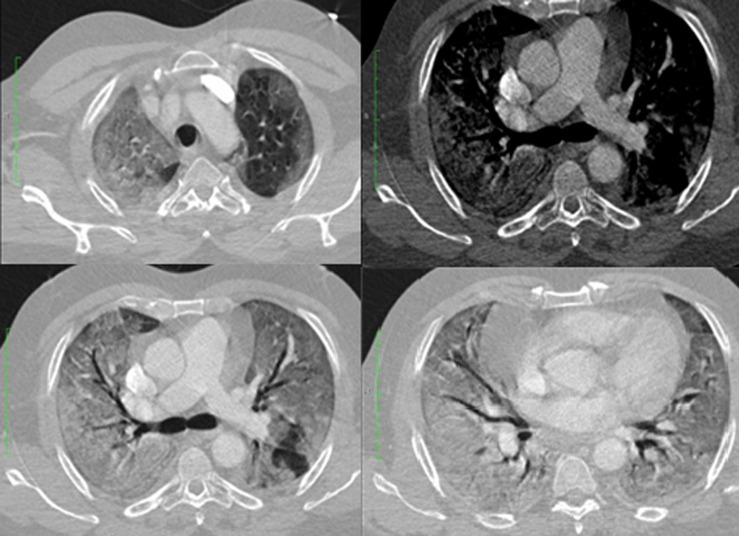
computed tomography images of case 3 on the 3^rd^ day of onset of symptoms showing bilateral lung consolidation (white lung) with a pan-lobar affectation and air-bronchograms; peripheral ground-glass opacification in the left upper lobe; no evidence of pulmonary embolism was seen

**Case 3:** was admitted on account of cough and difficulty in breathing of 3 days duration, initially exertional but later present at rest. He had no fever or chest pain. He was hypoxaemic and nursed in prone position with minimal improvements in the oxygen saturation. Arterial blood gasses showed respiratory alkalosis with compensatory metabolic acidosis. CRP and D-dimer were elevated. He had a CT pulmonary angiogram to visualize the lungs and rule out a pulmonary embolism. This showed bilateral alveolar consolidation with pan-lobar affectation, with typical radiological findings of adult respiratory distress syndrome (ARDS) Figure 3. Nasopharyngeal swab taken for RT-PCR testing for COVID-19 was positive. He was placed on mechanical ventilation and had no clinical improvement until his death less than 24 hours after (Figure 3). Computed tomography images of case 3 on 3^rd^ day of onset of symptoms showing bilateral lung consolidation (white lung) with a pan-lobar affectation and air-bronchograms. Peripheral ground-glass opacification in the left upper lobe. No evidence of pulmonary embolism was seen.

**Figure 4:**
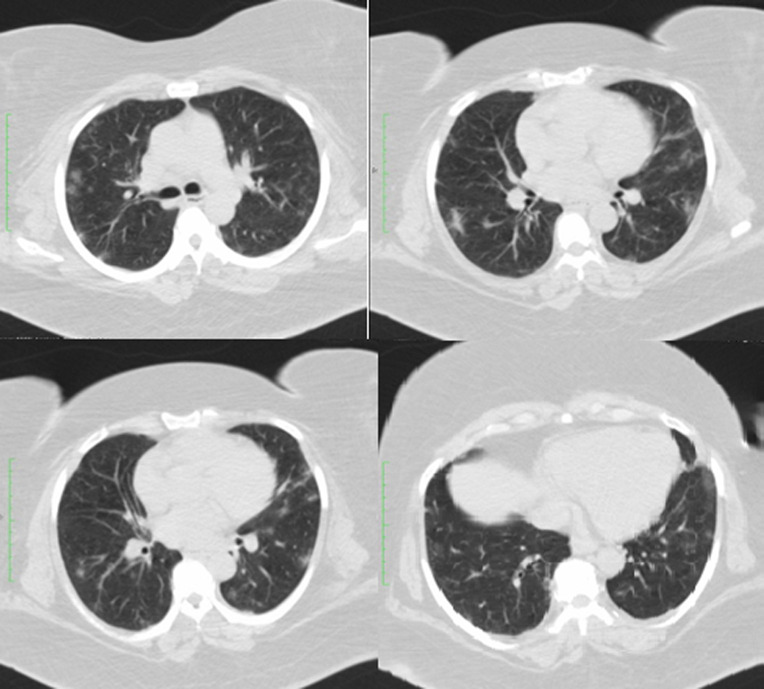
computed tomography scan images of case 4 on the 5^th^ day of symptom-onset, showing bilateral, nodular, ground-glass opacities in the upper and lower lobes with a peripheral distribution

**Case 4:** a female diabetic was admitted on account of weakness, diarrhoea and fever of 3 days duration. She had hyperglycaemia with a random blood glucose level of 347 mg/dl. She was desaturating in room air with SpO2 ranging between 88% and 94%. This also improved on nursing prone. A CT scan revealed bilateral, nodular, ground-glass opacities in the lower lobes with a peripheral distribution. RT-PCR results for COVID-19 taken on the 3^rd^ day on admission were positive. D-dimer and CRP were elevated. She was also evacuated to an isolation centre for further management and has since recovered (Figure 4).

## Discussion

COVID-19 is an infectious respiratory disease caused by the novel coronavirus. Its incubation period generally ranges from 1 to 14 days, with most people developing symptoms between 3-7 days [4]. The clinical severity of COVID-19 varies greatly, from asymptomatic to death. Mild cases mainly present with fever, fatigue, and a dry cough and atypically with diarrhoea and myalgia, while others may develop severe pneumonia, ARDS and multiple organ failure progressing to death [5]. Three of our four patients were males, consistent with studies reporting a higher incidence of COVID-19 in males [5, 6]. Fever, cough and difficulty in breathing were reported by our patients and fever and cough are consistent with the most common presenting symptoms reported in other studies with dyspnoea far less common [1, 5, 6]. Two of the patients also had diarrhoea and one reported chest pain, which are also recognised symptoms of COVID-19 [1, 3, 6].

Cases 2 and 4, with difficulty in breathing had oxygen saturations of 90% or less despite oxygen supplementation and this improved moderately on nursing prone. In index case 3, the clinical condition worsened and progressed to death despite mechanical ventilation. Hypoxaemia is reported to provide a robust risk factor for fatal outcomes and is associated with death in patients with COVID-19-associated pneumonia independently of age and sex [7]. It is also associated with higher neutrophil counts, D-dimer and CRP levels as well as acute inflammation of the respiratory system, caused by respiratory virus or secondary bacterial infection [7]. CRP and D-dimer were elevated in the more severe 3 of our index cases, also consistent with a moderate to severe inflammatory process [8]. Imaging for COVID-19 pneumonia mainly comprises chest radiography and computed tomography (CT). Our patients underwent X-rays and computed tomography scans. Chest radiographs are an initial and quick method of evaluating for significant lung abnormality. They may be less sensitive than CT, but are widely available, cost-effective and are suitable for primary hospitals which do not have CT machines as well as for the bedside examination of critically ill patients. Where there is high clinical suspicion of COVID-19, it is possible that a positive CXR may obviate the need for a CT [9]. Up to 89% of X-rays in COVID-19 may be interpreted as normal or mildly abnormal [10]. This is similar to findings in our patients as the X-ray in case 1 was normal while the chest X-ray in case 2 showed ill-defined bilateral alveolar consolidation with a peripheral and lower lobe distribution. It also excluded other pathology such as pneumothorax or pleural effusion. The other two cases did not have chest X-rays.

CT is the gold-standard for detecting the presence of lesions in the lungs, especially high-resolution CT, as it has no overlapping structural interference and can detect small lesions. It is useful for the diagnosis and differential diagnosis of COVID-19, monitoring treatment outcomes and early detection of other complications [3]. In the early stage, it shows multiple small patchy ground glass and interstitial changes, then develops into multiple ground glass shadows and infiltration with a peripheral distribution [3, 8], similar to the changes seen in our index cases 2 and 4. Ground-glass opacification is a slightly higher density, usually-rounded and blurred lesion in the lungs, where the pulmonary blood vessels are visible [1]. The ground-glass and/or consolidative opacities are usually bilateral, peripheral, and basal in distribution [1, 11]. This may be explained by viral invasion and replication in the bronchioles and alveolar epithelium causing inflammation and thickening of the alveolar wall with a distribution mainly around the lung and under the pleura [3]. In more severe cases, pulmonary consolidation may occur. Consolidation may be related to acute diffuse alveolar injury, including oedema, red blood cells and cellulose deposition. Thickening of the pulmonary interstitium or fibrosis may also be seen as stripes [3]. Pleural effusion and pneumothorax are rare [4]. Our cases run the gamut from mild to moderate to severe. Case 1 was a mild type with mild clinical symptoms and imaging findings showed no features of pneumonia. The chest CT manifestations of COVID-19 often presented patchy ground-glass opacities or mixed ground-glass opacities and consolidation, predominantly involving the periphery of both lungs, that can change rapidly [8]. Li *et al*. in a multicentre study in China showed that some patients with normal chest CT imaging could test positive for COVID-19 [8]. Cases 2 and 4 were of moderate severity with fever, cough, hypoxia and bilateral peripheral infiltrates, predominantly in the lower lobe in case 2. These findings are consistent with the findings described in other studies as the disease progresses [3, 8].

Case 3 was in the severe stage with shortness of breath and hypoxemia with typical radiological findings of ARDS, requiring mechanical ventilation with no relief until his death. As inflammation progresses, there is extensive involvement of alveoli, followed by consolidation. With a strong reaction to an inflammatory storm, large exudation occurs in the alveoli of both lungs, showing a white lung appearance [3, 11], as in the index case 3. Air bronchograms, also present in this patient, are the low-density shadowing of air-containing bronchus in the consolidation of lung tissue. This results from pathogenic invasion of the epithelial cells, causing inflammatory thickening and swelling of the bronchial wall without obstructing the bronchioles [3]. Other computed tomography features which have been documented in COVID-19 patients, but not seen in our index cases include paving stones sign, fibrosis, traction bronchiectasis, vascular thickening, halo or reverse halo signs [3].

## Conclusion

COVID-19 is a highly contagious disease with a wide range of severity of its clinical features. Imaging, both X-rays and CT are useful where there is suspicion of disease, for diagnosis, to rule out other conditions as well as for follow-up. These should be evaluated in conjunction with clinical and epidemiological features and laboratory investigations for early detection, isolation and treatment as required.

### What is known about this topic

- COVID-19 is an ongoing pandemic with rising numbers globally;
- Few studies in African populations have been published that document the clinical and imaging findings of COVID-19 patients.

### What this study adds

- More and more literature on COVID-19 is currently being published worldwide and this study will contribute to the growing knowledge;
- This study shares our spectrum of chest imaging features of COVID-19 and its management in a sub-Saharan African setting.
